# Physiologically relevant microsystems to study viral infection in the human liver

**DOI:** 10.3389/fmicb.2022.999366

**Published:** 2022-09-28

**Authors:** Dennis McDuffie, David Barr, Ashutosh Agarwal, Emmanuel Thomas

**Affiliations:** ^1^Department of Biomedical Engineering, University of Miami, Coral Gables, FL, United States; ^2^Department of Pathology and Laboratory Medicine, University of Miami Miller School of Medicine, Miami, FL, United States; ^3^Desai Sethi Urology Institute, University of Miami Miller School of Medicine, Miami, FL, United States; ^4^Sylvester Comprehensive Cancer Center, University of Miami Miller School of Medicine, Miami, FL, United States; ^5^Schiff Center for Liver Diseases, University of Miami Miller School of Medicine, Miami, FL, United States

**Keywords:** hepatitis B virus, hepatitis C virus, hepatocellular carcinoma, liver-on-chip, *in vitro* disease model

## Abstract

Viral hepatitis is a leading cause of liver disease and mortality. Infection can occur acutely or chronically, but the mechanisms that govern the clearance of virus or lack thereof are poorly understood and merit further investigation. Though cures for viral hepatitis have been developed, they are expensive, not readily accessible in vulnerable populations and some patients may remain at an increased risk of developing hepatocellular carcinoma (HCC) even after viral clearance. To sustain infection *in vitro*, hepatocytes must be fully mature and remain in a differentiated state. However, primary hepatocytes rapidly dedifferentiate in conventional 2D *in vitro* platforms. Physiologically relevant or physiomimetic microsystems, are increasingly popular alternatives to traditional two-dimensional (2D) monocultures for *in vitro* studies. Physiomimetic systems reconstruct and incorporate elements of the native cellular microenvironment to improve biologic functionality *in vitro*. Multiple elements contribute to these models including ancillary tissue architecture, cell co-cultures, matrix proteins, chemical gradients and mechanical forces that contribute to increased viability, longevity and physiologic function for the tissue of interest. These microsystems are used in a wide variety of applications to study biological phenomena. Here, we explore the use of physiomimetic microsystems as tools for studying viral hepatitis infection in the liver and how the design of these platforms is tailored for enhanced investigation of the viral lifecycle when compared to conventional 2D cell culture models. Although liver-based physiomimetic microsystems are typically applied in the context of drug studies, the platforms developed for drug discovery purposes offer a solid foundation to support studies on viral hepatitis. Physiomimetic platforms may help prolong hepatocyte functionality in order to sustain chronic viral hepatitis infection *in vitro* for studying virus-host interactions for prolonged periods.

## Key points

- Viral hepatitis is a significant contributor to hepatocellular carcinoma and liver disease.
- The transition of chronic infection to hepatocellular carcinoma and liver disease is poorly characterized and requires mechanistic investigation.
- Standard *in vitro* models are ineffective for studying this transition because hepatocytes do not maintain a functional phenotype that is permissive to infection long enough to model infection chronically.
- Physiomimetic microsystems could help bridge the gap to longer *in vitro* infections by incorporate elements of the hepatic microenvironment to promote functional longevity of hepatocytes.
- Physiomimetic models vary substantially, and draw on different elements of the hepatocyte microenvironment to sustain an infection-permissive phenotype most notably: Co-culture with non-parenchymal cells, 3D morphology, a physiological spatial orientation, and media perfusion.
- Physiomimetic models that were not originally designed to model viral hepatitis specifically may be used in chronic viral applications as long as they maintain hepatocyte functionality over a longer period of time than is possible in conventional 2D plate culture.

## Introduction

Hepatitis viruses are part of a select group of viruses that can establish chronic infections in humans. While most are quickly cleared after acute infection, hepatitis viruses B (HBV), C (HCV), D (HDV), and E (HEV) can all manifest as chronic infections (Dustin et al., 2016; Price, 2016). The mechanisms through which these infections transition from acute to chronic are poorly understood and merit further investigation into the relevant virus-host interactions. Chronic viral hepatitis infection is a leading cause of hepatocellular carcinoma (HCC), accounting for 1.3 million deaths per year and 90% of primary liver cancer cases (Ringehan et al., 2017).

One reason the transition from acute to chronic viral infection has not been properly investigated is a lack of sufficient *in vitro* models. Conventional two-dimensional (2D) *in vitro* models are excellent for generating high throughput data, but fail to provide cells with surroundings that mimics their native physiologic microenvironment. Because of this disconnect between *in vitro* culture and the *in vivo* microenvironment, primary hepatocytes, the gold standard of *in vitro* hepatocyte models, suffer from a loss of functionality after extended culture periods *in vitro*. This loss of functionality inhibits accurate long-term study of host responses to infection, which are instrumental for providing insight into how the transition from acute to chronic infection occurs. Furthermore, hepatoma cell lines and iPSC-derived hepatocyte-like cells (HLCs) do not provide a fully differentiated hepatocyte phenotype and it is difficult to draw relevant biologic conclusions from the observed cellular responses to infection using these models.

Animal models are indeed capable of sustaining chronic viral infections and have been critical to our understanding of the viral hepatitis and its progression to liver disease. However, in the context of viral hepatitis, humans and chimpanzees are the only two known naturally hosts for HBV and HCV, which makes studying the virus in animal models incredibly challenging given the ethical concerns of non-human primate models. Non-human analogs of hepatitis viruses exist, but these are fundamentally different from HBV and HCV, and their naturals hosts are challenged with replicating a human’s immunological response (Berggren et al., 2020; Liu et al., 2021). Even genetically humanized mice, modified to be infectible by human viruses, are limited in their ability to generate a relevant immune responses that is critical to uncovering the mechanisms of disease progression (Louz et al., 2013; Thomas and Liang, 2016). A need for animal models persists because even the most complex *in vitro* models cannot replicate a systemic response to infection, but these animal models must be supplemented by human-specific *in vitro* models.

In an effort to better supplement animal models, physiologically relevant microsystems are a potential solution to insufficient *in vitro* models. The purpose of these microsystems is to capture elements of the native cellular microenvironment *in vitro*, in an effort to improve experimental results as compared to a conventional 2D plate format. In the liver specifically, much of the work in the development of physiologically relevant microsystems has been directed toward pharmacokinetic modeling and drug-induced liver injury. Nonetheless, many of the same principles used in these systems to improve cell functionality can be effectively utilized in modeling virus-host responses. In this review we investigate how physiologically relevant microsystems recapitulate liver physiology and also how this has led to advances in the study of viral hepatitis infection *in vitro*.

We first review the physiology of the liver and the necessary components required for relevant studies of viral hepatitis infection *in vitro*. We then examine different cell models for studies on viral hepatitis *in vitro*. Next, we provide an overview of a wide variety of practical designs of physiologically relevant microsystems that model the liver and discuss the applications of these models as tools to study virus-host interactions. Finally, we discuss some of the elements of physiologically relevant microsystems that enhance their ability to study viral hepatitis biology specifically, and how these systems are working toward better informing clinical predictions.

## Viral hepatitis and the liver

### Hepatic microenvironment

**FIGURE 1:**
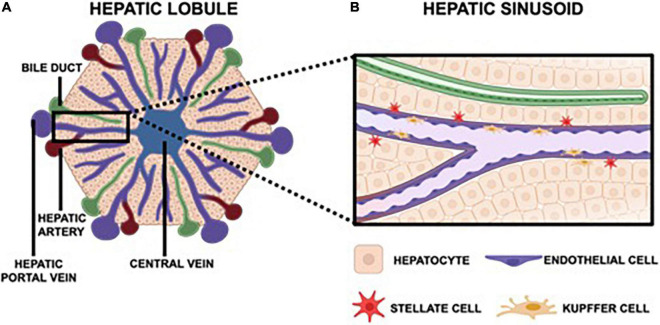
The hepatic lobule is hexagonal, functional unit of the liver that features six portal triads. Each portal triad is comprised of a bile duct, a hepatic portal vein, and a hepatic artery **(A)**. The hepatic portal vein and hepatic artery converge to form the hepatic sinusoid, which carries blood toward the central vein that then returns to the heart. Each sinusoid is lined with endothelial cells and surrounded by hepatocytes, the parenchymal cells of the liver. Also native to the sinusoid are Kupffer cells, the resident macrophages, and stellate cells, which are quiescent in a healthy liver but instigate a fibrotic response in an inflammatory microenvironment **(B)**.

By leveraging our current understanding of liver physiology and pertinent aspects of viral pathogenesis, one can ascertain the most critical components of the hepatic microenvironment needed to accurately recapitulate viral hepatitis infection *in vitro*. Overall, the hepatic lobule is the functional unit of the liver (Figure 1A). The lobule is a hexagonal structure comprised of six portal triads. Each portal triad includes a connection to a hepatic portal vein, a hepatic artery and a bile duct. The hepatic portal vein and the hepatic artery branch together to form the hepatic sinusoid, that is lined with endothelial cells and which transports blood from portal venules and hepatic arterioles (periportal sides) to the central vein (perivenous side) that eventually flows back to the heart. Interspersed between the liver sinusoids are plates of hepatocytes, the parenchymal cells of the liver, that are the substrate of viral hepatitis infection where the virus lifecycle is carried out (Ishibashi et al., 2009; Lautt, 2009; Rocha, 2012). A single sinusoid flanked by hepatocytes distinguishes the smallest functional unit of the liver, the hepatic acinus. The acinus also includes non-parenchymal cells (NPCs) namely Kupffer cells (KCs), the resident macrophages of the liver, and stellate cells (SCs) which are normally quiescent but become fibrogenic when activated by perturbations to the normal liver environment (Zheng et al., 2009; Deng et al., 2019a; Figure 1B). Both hepatocytes and NPCs contribute to the inflammatory microenvironment in response to viral hepatitis infection and are further implicated in the progression of disease as chronic infection persists (Feldstein et al., 2005; Dixon et al., 2013; Li et al., 2017; Hyun et al., 2019).

### Viral hepatitis

Viral pathogens that cause hepatitis are noteworthy in their ability to establish chronic infection. There are five major hepatitis viruses (A-E) each with several different genotypes. These viruses vary greatly; however, in this review, we focus on HBV and HCV due to their worldwide prevalence and strong link to significant morbidity and mortality.

#### Hepatitis B virus

HBV is a partially double-stranded DNA virus that produces high levels of viral antigens which enabled the development of a highly effective vaccines against the virus (Thomas and Liang, 2016). Although vaccines have proven successful in preventing the transmission of HBV, infection remains a challenge because of the lack of a highly effective, finite curative treatment regimen and also since chronic HBV infection has a clear link to the development of HCC (NIH strategic plan details pathway to achieving Hepatitis B cure, 2019; Torresi et al., 2019). Moreover, as of 2019, global coverage of the HBV vaccine birth dose was at just 42%; unfortunately 90% of babies will progress to chronic infection if infected via mother-to-child transmission (hepbtalk, 2019). After entering the hepatocyte, the virus translocates to the nucleus where its genome is modified to the covalently closed circular (cccDNA) that exists stably as an extrachromosomal genome and is a major viral component contributing to viral persistence. cccDNA codes for the transcripts necessary for protein production and replication; therefore, it is the primary target for potential curative therapies (Thomas and Liang, 2016). *In vitro* models to further characterize the progression of HBV infection from acute to chronic infection are desperately needed and also models that can be utilized to further develop effective curative regimens are highly sought after.

#### Hepatitis C virus

HCV is a single-stranded positive-sense RNA virus that manifests chronically in 75–85% of those infected (Chen and Morgan, 2006). HCV enters the hepatocyte and completes translation and replication in the endoplasmic reticulum (Thomas and Liang, 2016). In contrast to HBV, HCV is curable through treatment with small molecules that include polymerase and protease inhibitors that are more broadly referred to as direct acting antiviral agents (DAAs); unfortunately, there is no vaccine against HCV because of the virus’ high degree of genetic variability (Zingaretti et al., 2014). Furthermore, patients infected with chronic HCV remain susceptible to developing HCC even after the virus is cleared if they have advanced liver disease (El-Serag, 2002; de Oliveria Andrade et al., 2009; Axley et al., 2018; Ioannou et al., 2019; Nahon and Ganne-Carrié, 2019). Similar to HBV, the need for further study of HCV stems from gaps in knowledge of the virus’ molecular strategies that allow it to transition from acute to chronic infection and host factors that influence this biologic outcome.

## *In vitro* cellular models of viral hepatitis infection

Choice of hepatocyte source has a substantial impact on the biologic validity of experimental results as well as on the efficiency of supporting infection *in vitro*. Primary hepatocytes, hepatoma cell lines and iPSC-derived hepatocytes have significant differences in various experimental characteristics; however, each model may be desirable for use depending on the specific experimental application.

### Primary hepatocytes

Primary human hepatocytes (PHHs) are the gold standard for *in vitro* liver models in general. They display a notable differentiated phenotype at the time of plating. In addition, they support both HBV and HCV infection; unfortunately, their potent innate antiviral response is difficult to circumvent rendering this model unsuitable for the production of high titer virus stocks. In concordance with this, it is more difficult to establish long-term infections in these cells without artificially downregulating the antiviral response. Additionally, PHHs are a relatively rare cell source given that they come directly from patients or fetal tissue and they do not naturally divide once cultured *in vitro*. Inherent interpatient variability also drives the need to source cells from multiple donors to achieve more reproducible results (Thomas and Liang, 2016). However, humanized mice have recently been developed to propagate PHHs *in vivo* which has rendered these cells somewhat easier to source (Ohshita and Tateno, 2017).

### Hepatoma cell lines

Hepatoma cell lines provide a high degree of accessibility for modeling viral infection *in vitro* at the cost of being unable to provide normal physiologic antiviral responses. Here we describe three different cell lines frequently used to model viral hepatitis infection *in vitro*.

#### Huh7

The Huh7 cell line and its genetically modified successors are very receptive to HCV infection. These cells are excellent at propagating infectious HCV virions and they divide rapidly. Huh7 cell are extremely useful for replicating viral infection, and this cell line also supports infection with other important pathogenic viruses including SARS-CoV-2 (Thomas and Liang, 2016; Chen et al., 2021). However, Huh7 cells have significantly attenuated cell-intrinsic innate antiviral responses to viral infection, and therefore are not useful for studying the viral immune response in hepatocytes.

#### HepG2

HepG2 cells have been used for studies of the HBV lifecycle. HepG2 cells are polarized, can be manipulated to express the HBV genome and can generate a cell-intrinsic innate antiviral response that makes them useful for the studies on host defense pathways. However, HepG2 cells must be genetically modified in order to fully support HBV infection as they lack significant expression of the NTCP HBV viral entry receptor, and even when this receptor is expressed, HepgG2 cells are still less susceptible to HBV infection than fully differentiated HepaRG cells (Li et al., 2016; Thomas and Liang, 2016).

#### HepaRG

The HepaRG cell line is derived from a bipotent progenitor that can differentiate into either the hepatocyte or cholangiocyte lineage. If differentiated toward the hepatocyte lineage, HepaRGs confer a phenotype that mirrors that of PHHs in terms of its ability to support both HBV and HCV infection while demonstrating contact inhibition (Thomas and Liang, 2016; Wu et al., 2016). However, this differentiation process can take up to 2 weeks and the differentiated cells appear to be difficult to propagate further (Thomas and Liang, 2016). HepaRGs are also bipotent progenitor cells and therefore may lack a fully functional innate immune axis (Shlomai et al., 2014). Finally, HCV is extremely difficult to replicate in HepaRGs and to our knowledge, has only been achieved by Ndongo-Thiam et al. (2011).

### Stem cell derived hepatocytes

iPSC-derived hepatocyte-like cell (HLCs) are similar to HepaRGs in the sense that they can be differentiated in order to confer a phenotype that approaches that of PHHs; however, they have yet to prove capable of exhibiting the same level of differentiation as PHHs that includes minimal to no expression of alpha-fetoprotein. Unlike PHHs however, iPSCs offer a limitless supply of cells while minimizing donor variability. HLCs have demonstrated the capacity of supporting both HBV and HCV infection (Wang et al., 2019; Simoneau and Ott, 2020).

## Physiologically relevant liver technologies and their application to viral hepatitis

**FIGURE 2:**
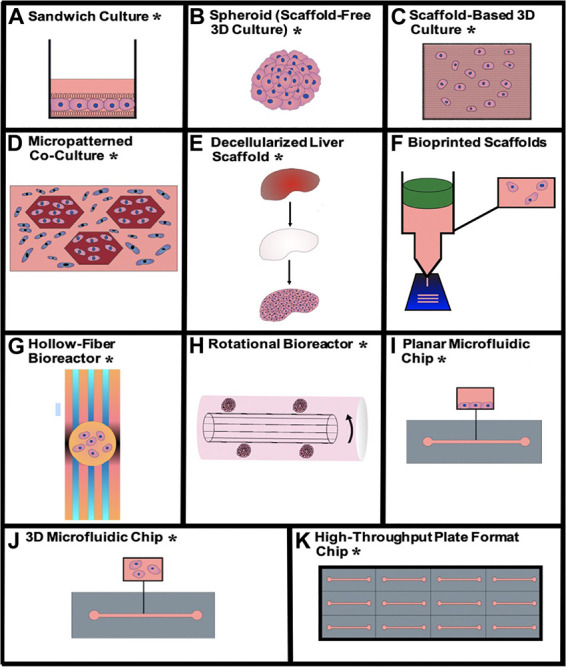
Visualizations of the different physiomimetic culture systems outlined in this review. **(A)** Sandwich culture*, **(B)** aggregate spheroid culture*, **(C)** scaffold-based 3D culture*, **(D)** micropatterned co-culture*, **(E)** decellularized liver scaffold*, **(F)** bioprinted scaffolds, **(G)** hollow-fiber bioreactor*, **(H)** rotational bioreactor*, **(I)** planar microfluidic chip*, **(J)** 3D microfluidic chip*, **(K)** high-throughput plate format chip*. *Indicates that the given type of model has been used for viral hepatitis study.

Physiomimetic *in vitro* liver models are typically designed with the goal of promoting and sustaining hepatocyte differentiation and viability and are then applied to study relevant functional biological mechanisms. This preliminary step of promoting the maintenance of viable and differentiated hepatocytes is critical for the establishment of a microenvironment that is conducive to support long-term viral infection. Here, we describe how different technologies recapitulate the hepatic microenvironment to best support and maintain physiologically relevant hepatocyte function. The advantages and disadvantages of each type of physiomimetic platform are listed in Table 1 and the different platforms are depicted in Figure 2.

**TABLE 1:** Comparative overview of different physiomimetic culture platforms.

Culture system	Advantages	Disadvantages
Static systems		
Sandwich culture	• High-throughput • Low maintenance • Accessible	• Overly simplistic • Minimal benefits beyond traditional 2D culture
Spheroids	• 3D cell orientation • High-throughput • Low maintenance	• Non-physiological microenvironment • Necrotic cores • Variability between spheroids
3D scaffolds	• 3D cell orientation • High-throughput	• Variable nutrient exchange (depending on scaffold properties) • Development of necrotic regions
MPCCs	• Excellent longevity • High-throughput • Low maintenance	• Non-physiological microenvironment • Incorporates non-human cells
Decellularized scaffolds	• Provide a physiological microenvironment • Versatile applications • 3D cell orientation	• Difficult to source • Extensive preparation and characterization required
Bioprinted scaffolds	• High degree of customizability • Can recapitulate hepatic architecture • 3D cell orientation	• Time intensive • Requires bioprinting equipment
Perfusion systems		
Hollow-fiber bioreactors	• Provide a physiological microenvironment • 3D cell orientation • Excellent longevity	• Large • Low-throughput • Low cell accessibility
Rotational bioreactors	• 3D cell orientation • Excellent longevity	• Low-throughput • Low cell accessibility
Planar perfusion chips	• Provide physiological shear and nutrient exchange • Can recapitulate hepatic architecture • Small device	• Non-physiological cell orientation • Difficult to prepare
3D Perfusion chips	• 3D cell orientation • Provide physiological nutrient exchange • Can recapitulate hepatic architecture • Small device	• Difficult to induce fluid-driven shear when cells are encapsulated • Difficult to prepare
High-throughput perfusion chips	• High-throughput • Amenable to large number of conditions • Can recapitulate hepatic architecture • Provide physiological nutrient exchange	• Difficult to prepare

### Static culture models

Static models established the foundation for the subsequent development of dynamic models and they are utilized for the development of tissue-like structures beyond traditional 2D cell monolayers. Any fresh media that the culture receives has to be administered manually and these cultures are characterized by the lack of any consistent media perfusion or flow through the model.

#### Sandwich culture

Sandwich culture is a layered culture approach where cells are sandwiched between two matrix layers (Figure 2A). Cells bind and form a monolayer on the matrix beneath them and are subsequently coated with a top layer of matrix that is called an overlay. This physiomimetic sandwiched orientation has been shown to improve cell morphology, viability and maintenance of differentiation that includes the establishment of polarity and formation of bile canalicular networks (Swift et al., 2010; Yang et al., 2016). Complexity of these cultures increases even further in co-culture sandwiches. Mouse fibroblasts (3T3-J2) were used as feeder cells to help differentiate iPSCs into HLCs as double sandwiches where the fibroblasts were stacked on top of the hepatocytes (Nagamoto et al., 2012). Sasaki et al. (2017) performed a layer-by-layer (LBL) cell coating of hepatocytes and fibroblasts on a membrane with distinct fibronectin and gelatin layers. They subsequently developed a tri-culture with human umbilical vein endothelial cells (HUVECs) to create a vascularized tissue. Petropolis et al. (2016) implemented this vascularized LBL approach, specifically for studies on HBV replication, through the formation of two Huh7 hepatocyte layers separated by a matrix and topped with a layer of endothelial cells. The establishment of sandwich cultures were an important advance in establishing more physiologically relevant models and they remain the foundation of more complex physiomimetic platforms.

#### 3D spheroids, organoids, and scaffolds

3D spheroids, organoids, and scaffolds are techniques for culturing cells in 3D by providing cells with points of contact in three dimensions to further support the maintenance of hepatocyte differentiation *in vitro* (Bachmann et al., 2015). Though ease of use has precluded the full transition from 2D cultures to these less complex 3D cultures for *in vitro* work, 3D cultures may promote more stable long-term cell function that would be appropriate for extended viral infection studies.

##### Scaffold-free 3D culture

Cell aggregates can create a scaffold-free 3D cultures (Figure 2B). Cells attach to each other to form multi-cellular spherical structures called spheroids, or organoids if there are multiple cell types. The simplest method of spheroid or organoid formation is through the use of ultra-low attaching (ULA) surfaces, that are treated to keep cells in suspension, causing them to aggregate into spheroids (Subramanian et al., 2013; Kim et al., 2015; Miyamoto et al., 2015; Gaskell et al., 2016; Hendriks et al., 2016; Ramaiahgari et al., 2017; Vorrink et al., 2017, 2018; Bell et al., 2018; Kozyra et al., 2018; Mandon et al., 2019). These plates can also be used for co-cultures or multi-lineage differentiation to form hepatocyte organoids (Bin Ramli et al., 2020). Hanging-drop plates are an alternative method of spheroid generation that use hanging drops of small volumes of cell suspension to allow hepatocytes to aggregate over 2–4 days before being transferred to a new plate for experimentation (Gunness et al., 2013; Mueller et al., 2014; Takahashi et al., 2015). This technique has been commercialized by InSphero and is also applicable to organoid co-cultures (Messner et al., 2013, 2018; Proctor et al., 2017). Nie et al. (2018) generated organoids from iPSC-derived HLCs and HepG2s each of which were co-cultured with HUVECs. They used these iPSC-derived organoids to study cellular interactions with HBV for up to 20 days post-infection, with results comparable to 2D PHH culture. Spheroids and organoids appear to be useful tools for simple 3D cell culture applications; however, they lack an organized physiological architecture and they may also suffer from the establishment of necrotic cores due to limited oxygen concentration within the center of the structure (Bachmann et al., 2015).

##### Scaffold-based 3D culture

Scaffold-based approaches for 3D hepatocyte culture typically employ hydrogel matrices for bulk encapsulation (Figure 2C). These products are available in multiple forms that includes Matrigel^®^, Geltrex, PuraMatrix, alginate, chitosan, gelatin/collagen, polyethylene glycol diacrylate (PEGDA) and cellulose where the porous structures support the transport of cell signaling molecules between hepatocytes (Barbetta et al., 2008; Ramasamy et al., 2013; Gieseck et al., 2014; Ramaiahgari et al., 2014; Ramachandran et al., 2015; Sirenko et al., 2016; Tasnim et al., 2016; Wang et al., 2016; Nuciforo et al., 2018; Ahn et al., 2019; Mun et al., 2019; Ouchi et al., 2019). Crignis et al. (2020) infected healthy donor organoids suspended in a basement membrane matrix with HBV and demonstrated HBV cccDNA production up to 8 days post-infection. Cho et al. (2008), Molina-Jimenez et al. (2012), and Ananthanarayanan et al. (2014) embedded cells in Matrigel, PEGDA hydrogel and a cellulose hydrogel, respectively, followed by subsequent infection of the spheroids with HCV. Similarly, Rajalakshmy et al. (2015) cultured Huh7 cells in Mebiol gel, a thermoreversible gelatin polymer, and infected them with HCV for 10 days. In addition to bulk encapsulation, cells can also be fractionated into small beads (Selden et al., 2000; Khalil et al., 2001; Hwang et al., 2010; Huch et al., 2015; Rebelo et al., 2015; Phillips et al., 2018; Nakai et al., 2019). For example, Tran et al. (2012) used an optimal Ca-Na-alginate bead formulation to upregulate expression of HCV-specific receptors in Huh7 cells, rendering the model useful for HCV infection studies. Substrate mechanical properties do impact hepatocyte function; therefore, scaffolds and matrices can be supplemented and tuned to match desired *in vivo* mechanical properties (Matellan and Del Río Hernández, 2019). Non-hydrogel scaffolds like paper and nylon have also been used to provide a consistent rigid structure while the additional use of tunable cross-linking of hydrogels can be leveraged to modulate mechanical properties *in situ* (Kostadinova et al., 2013; Zhu et al., 2017; Wang et al., 2018a). Akahori et al. (2020) used a silica fiber scaffold to support HBV cccDNA formation, in 3D hepatocytes, for up to 15 days post-infection. Depending on the material of choice, 3D rigid scaffolds offer the potential of a more mechanically relevant microenvironment compared to scaffold-free cultures (Matellan and Del Río Hernández, 2019).

#### Micropatterned co-cultures

Micropatterned co-cultures (MPCCs) are deterministically patterned co-cultures, often achieved by selective matrix deposition or surface treatment (Figure 2D). The original hepatocyte MPCC design from Khetani et al. (2013) now commercialized as HEPATOPAC^®^, features groups of hepatocytes seeded on collagen, spatially arranged via stencil, and surrounded by 3T3-J2 mouse fibroblasts as feeder cells (Khetani and Bhatia, 2008; Wang et al., 2010; Khetani et al., 2013; Trask et al., 2014; Ware et al., 2015; Aleo et al., 2019; Chan et al., 2019). Like sandwich cultures, HEPATOPAC^®^ offers many of the same advantages as 2D culture in terms of accessibility and throughput translating seamlessly to 24, 96, and 384-well plate formats (Ware et al., 2015; Gural et al., 2018). This patterning with feeder cells improves the longevity and functionality of both PHHs and iPSC-derived hepatocytes when compared to hepatocyte monoculture (Berger et al., 2015; Ware et al., 2017). Ploss et al. (2010) co-cultured cultured PHHs with 3T3-J2s in the HEPATOPAC^®^ system and achieved HCV infection for up to 2 weeks. Similarly, Shlomai et al. (2014) used MPCC technology to culture PHHs with 3T3-J2s; however, they used successful HBV infection as a metric of functional differentiation of MPCCs that was not limited exclusively to co-culture of hepatocytes with 3T3s. The system also features the possibility of co-culturing hepatocytes with stellate cells, Kupffer cells and endothelial cells for the purpose of improving the maintenance of hepatocyte function (Nguyen et al., 2015; Davidson et al., 2017; Ware et al., 2017). Micropatterning can also be accomplished using spheroids as opposed to hepatocytes in a monolayer (Fukuda et al., 2006). The Cell-Able^®^ plate features attaching and non-attaching regions that are produced through the use of a photo-sensitive material. This material allows for spatial patterning of self-assembling hepatocyte spheroids that can then be cultured with or without 3T3 cells (Ohkura et al., 2014; Ogihara et al., 2015). In addition to surface-guided patterning, segregated flow techniques have been used to pattern hydrogel co-cultures in 3D. Kobayashi et al. (2013) used a patterned chip design, featuring an alginate spacer, to separate hepatocytes and 3T3 cells both of which were suspended in distinct alginate solutions. Similarly, Jia et al. (2020) created a hepatic plate using alternating layers of hepatocyte and endothelial alginate solutions^159^. Using a creative technique to avoid flow through separate channels, Ho et al. (2013) flowed cell suspensions into a chip and used dielectrophoresis to guide cells into a radial pattern that mimics the structure of a lobule using hepatocytes and endothelial cells. Kukla et al. (2020) used matrix encapsulation of hepatocytes and feeder cells to generate 3D co-encapsulated microtissues, which enhanced hepatocyte function. Overall, MPCCs provide spatial control over co-cultures to optimize the synergistic effects between different cell types while maintaining the differentiated hepatocyte phenotype that is necessary for infection. It is important to note that for studies that are specifically focused on understanding human disease processes, the presence of murine feeder cells may render the system suboptimal given the possible non-physiologic contributions to observed experimental outcomes. The effect of the feeder cells may be minimized by their selective killing before proceeding with experimentation on the remaining hepatocytes.

#### Decellularized scaffolds

Decellularized scaffolds from both human and non-human livers provide a physiological matrix via preservation of liver-specific ECM (Figure 2E). This native ECM supports requisite signals for engraftment, survival and function following hepatocyte reseeding (Rossi et al., 2019). Liver tissue is first decellularized by perfusion of detergents and/or enzymes leaving only matrix proteins behind. The matrix foundation is then re-seeded with hepatocytes and any other NPCs for the purpose of extending and improving hepatocyte function (Baptista et al., 2011; Lang et al., 2011; Wang et al., 2011; Mazza et al., 2015; Maghsoudlou et al., 2016). Zhang et al. (2019) seeded healthy and cirrhotic patient decellularized liver ECM (dECM) scaffolds with HBV-infected HepG2-NTCP cells. The authors found greater viral replication in the scaffold from the cirrhotic patient. Furthermore, when compared to 2D HBV infection, viral replication was markedly increased even in the healthy scaffold. These findings indicate that the liver microenvironment may have a significant impact on viral infection *in vitro* (Zhang et al., 2019). dECM proteins can also be preserved independent of maintaining the liver microarchitecture. The decellularized scaffold can be pulverized into a raw ECM protein suspension for subsequent manipulation and use for surface coating, spheroid formation and as bioink in 3D printing applications (Bavli et al., 2016; Mi et al., 2018; Yajima et al., 2018; Deng et al., 2019b). The major drawback of dECMs is their low degree of accessibility relative to other platforms that includes the cumbersome methods of preparation required to fully decellularize and validate the decellularization of the liver substrate (Maghsoudlou et al., 2016). However, based on efficacy alone, decellularized scaffolds are a promising mimetic of the *in vivo* microenvironment. This material has the potential for use within multiple applications, both as a standalone model and also as an enhancement to the physiologic performance of other *in vitro* liver models.

#### Bioprinted scaffolds

3D bioprinting is a novel technology that incorporates biological materials into 3D printing that allows for fine spatial control to form physiologically relevant 3D tissue architecture via layer-by-layer deposition that can lead to improved maintenance of hepatocyte function (Ma X. et al., 2016; Figure 2F). Bioinks are typically hydrogel-based that can be crosslinked into a more rigid structure post-printing. However, as mentioned previously, bioinks can be synthesized from dECM providing a more physiologically accurate protein composition (Skardal et al., 2015; Lee et al., 2017; Mao et al., 2020). The spatial arrangements of bioprinted scaffolds vary substantially in terms of complexity. Scaffolds printed from single bioinks are functionally similar to traditional 3D scaffolds in their capacity to improve long-term viability and maintenance of phenotypic differentiation (Faulkner-Jones et al., 2015; Jeon et al., 2017; Grix et al., 2018). For co-cultures, however, bioprinting is advantageous over traditional scaffolds because it can incorporate multiple bioinks containing different cell types. These different bioinks can then be used to manufacture deterministically patterned multi-cellular vascularized sinusoid-mimetic constructs (Matsusaki et al., 2013; Ma X. et al., 2016; Nguyen et al., 2016; Wu et al., 2018; Taymour et al., 2021). Using separate bioinks for different cell types also allows for the creation of a physiologic separation of different cell types while still permitting cellular cross-talk. In the case of bioprinting a liver, hepatocytes are often separated from NPCs, as they are *in vivo*, through separately deposited bioinks. Hiller et al. (2018) published a combinatory alginate-dECM bioink that demonstrated promise for viral studies based on its permissiveness to adenoviral replication; however, they did not infect cells in their system with HBV or HCV. Nonetheless, bioprinting holds great potential for creating more relevant *in vitro* models because of its high degree of spatial control and customizability for a given experimental application.

#### Novel 3D culture techniques

Alternative 3D culture methods have been developed to optimize cell accessibility, viability, spatial distribution and experimental throughput. Self-organizing organoids have been generated from stem cell precursors, both by using by using endothelial cell co-culture to modulate interactions with stem cells, and by overexpressing the GATA6 gene in a custom-engineered stem cell line (Takebe et al., 2013; Guye et al., 2016; Velazquez et al., 2020). Desai et al. (2017) used magnetic nanoparticles that attached to cell membranes and then used magnets to aggregate the cells to form spheroids. Miyamoto et al. (2015) and Ott et al. (2017) used similar micromolded well designs where cells congregated and formed spheroids due to gravity. Wang et al. (2015) used an RGD/galactose conjugated surface to form tethered spheroids whereas Chen et al. (2016) utilized a water/water/oil emulsion to create a complex spheroid with a hepatocyte-laden core and a 3T3-J2 mouse fibroblast shell. Micropillar and nanopillar technology has proven effective for high throughput studies through minimizing the use of space. The pillars provide a location for 3D cell or matrix attachment and also facilitate experiments that require exposure to various compounds or other treatment conditions (Takayama et al., 2013; Kwon et al., 2014; Roth et al., 2018).

### Perfusion culture models

Perfusion cultures subject cells to consistent laminar flow of media. These platforms use the building blocks of static culture to establish a more complex dynamic culture that may provide physiologically relevant mechanical stimulation and nutrient exchange. Healthy mechanical stimulation and nutrient exchange promotes prolonged cell viability and function to a degree that may otherwise be unattainable when utilizing static cultures (Kalyanaraman et al., 2008; Huh et al., 2011).

#### Bioreactors

Bioreactors are advanced technologies that established the foundation for the subsequent development of microfluidic chips. The purpose of bioreactors is to recapitulate an isolated system *ex vivo*. Bioreactors are amenable to long-term culture of tissues but are less useful as models for high-throughput screening when compared to their miniaturized microfluidic chip counterparts (Tostões et al., 2012).

##### Hollow fiber bioreactors

Hollow fiber (HF) bioreactors are perfusion devices that typically feature two sets of hydrophilic media capillaries and one set of hydrophobic oxygen capillaries (Figure 2G). The media capillaries provide counter-current flow for nutrient exchange while the oxygen capillaries promote gas exchange. Cells are cultured in 3D compartments between the HF capillaries (Darnell et al., 2012). Traditionally, HF bioreactors have been used to prolong the functional lifespan of primary hepatocytes for extended studies *ex vivo* (Pless et al., 2006; De Bartolo et al., 2009; Schmelzer et al., 2009; Zeilinger et al., 2011; Hoffmann et al., 2012; Ulvestad et al., 2012; Lübberstedt et al., 2015; Ahmed et al., 2017). Gerlach et al. (2003) maintained PHHs and NPCs for 5 weeks. In a separate study, Aizaki et al. (2003) sustained HCV infection in the FLC4 cell line for over 100 days in their HF bioreactor. More recently, these bioreactors have been utilized as a platform for differentiating stem cells or progenitor cell lines to HLCs (Kawada et al., 1998; Sivertsson et al., 2012; Meier et al., 2017). Though useful for supporting hepatocyte differentiation, HF bioreactors lack the scalability and throughput capacity that are desired for other types of viral infection studies that require biologic or technical replicates.

##### Rotational bioreactors

Rotational bioreactors, or rotating wall vessels (RWVs), are platforms for spheroid generation and/or maintenance that continuously suspend cells and allow them to aggregate into spheroids (Yoffe et al., 1999; Chang and Hughes-Fulford, 2008; Leite et al., 2012; Figure 2H). The spheroid cultures can be maintained in the RWV or removed for encapsulation/experimentation after they are generated (Fey and Wrzesinski, 2012; Jung et al., 2017). Rebelo et al. (2017) successfully formed an organoid with a parenchymal cell core and stromal cell shell, without emulsions, by allowing hepatocyte spheroids to aggregate first before introducing BM-MSCs that coat the hepatocyte spheroids on the outside. Sainz et al. (2009) used an RWV to culture and infect Huh7 spheroids for up to 2 weeks with HCV. RWVs are a good option for long-term spheroid culture, but like HFs, they translate poorly to support high throughput studies.

#### Microfluidic platforms

Microfluidic platforms are the miniaturized successors to bioreactors that possess numerous advantages including easier multiplexing, imaging and sample collection. Furthermore, it leverages the use of fewer reagents and cells in its layout to better mimic the liver’s architecture. These microfluidic platforms are often designed as perfusion analogs to the static models delineated above. Liver microfluidic devices are most commonly fabricated from PDMS elastomer, PMMA (acrylic), or polystyrene. They can also incorporate numerous other materials in their design that can be manufactured with a wide variety of formfactors. Despite their differences in design and means of construction, these devices are ultimately unified in their aim to better capture the physiology of the liver *in vitro*.

##### Planar and layered microfluidic platforms

As a perfusion analog to monolayers and sandwich culture, planar and layered microfluidic platforms expose a monolayer or multiples layers of cells to laminar flow (Figure 2I). This laminar flow profile is generally consistent with blood flow through the sinusoid, thus providing the culture with simulated physiological shear and nutrient exchange (Natarajan et al., 2017). Planar flow cultures are categorized by two different techniques for cell arrangement: Membrane/barrier cultures and non-membrane cultures. Membrane and barrier cultures create a boundary that can separate different cell types or selectively regulate flow across cells in order to modulate nutrient transport (Lee et al., 2007; Carraro et al., 2008; Zhang et al., 2008; Illa et al., 2014; Maher et al., 2014; Rennert et al., 2015; Prodanov et al., 2016; Ylva et al., 2016; Ortega-Ribera et al., 2018; Deng et al., 2019b). Non-membrane cultures feature some permutation of cells and ECM layers with no physical barrier to separate cells from each other or from flow through the system (Leclerc et al., 2004; Baudoin et al., 2011; Prot et al., 2011, 2014; Cheng et al., 2012; Choucha Snouber et al., 2013; McCarty et al., 2014; Giobbe et al., 2015). Membrane/barrier cultures better mimic the liver’s architecture; however, cell accessibility may be more limited for imaging and sample collection and cell-cell signaling between different cell types in co-culture is likely encumbered by a more substantial physical barrier when compared to ECM layered cultures. Conversely, non-membrane cultures require a less complex chip design with easier cell access, but fail to recapitulate the physical barrier between hepatocytes and the linear path of flow that exists *in vivo*. Without this barrier, hepatocytes are at risk of exposure to non-physiological shear that may affect the long-term maintenance of a differentiated phenotype. Emulate’s Liver-Chip features a membrane that divides dual parenchymal and non-parenchymal perfusion channels separated by a PDMS membrane with individual flow rate control that is amenable to imaging (Foster et al., 2019; Jang K. J. et al., 2019; Peel et al., 2019). Kang et al. (2017) also used a bi-layer membrane chip design that was able to support HBV infection in PHHs for 14 days. Alternatively, HμREL employed a membrane-free approach using PHHs seeded and perfused in monoculture or co-culture with NPCs on a collagen coated polystyrene chip (Chao et al., 2009; Novik et al., 2010; Hultman et al., 2016). Several planar chips, membrane and non-membrane alike, have attempted to recreate the natural zonation of the liver via oxygen tension characterization and concentration patterning of metabolic modulators (Lee-Montiel et al., 2017; Kang et al., 2018; Li et al., 2018; Moya et al., 2018; Tonon et al., 2019). Zonation chips may provide a more holistic perspective when studying the liver. Banaeiyan et al. (2017) developed a barrier model to study the liver at an expanded scale through recreating 18 full liver lobules as opposed to a single sinusoid. Compatible with both planar and ECM encapsulated culture, this dual layer chip incorporates diffusion barriers that separate sections of hepatocytes to mimic the shear-protective and nutrient-modulating endothelial layer. Merging planar cultures with laminar flow is a promising technique for maintaining a differentiated hepatocyte phenotype that can sustain viral infection.

##### 3D microfluidic platforms

3D microfluidic devices are acutely similar to planar microfluidic devices by design, but provide a difference in cell orientation. Like static 3D cultures, cells are cultured as scaffold-free spheroids/organoids, individual scaffold-encapsulated 3D cells, or scaffold-encapsulated spheroids/organoids. Scaffold-free cultures require physical barriers in the form of micropillars or protective membranes to immobilize and protect spheroids from being perturbed by direct exposure to flow (Goral et al., 2010; Ong et al., 2017; Wang et al., 2018b). In Ma et al., 2018 design, the chip was manufactured with microwells where spheroids congregate, topped with a microporous membrane to simulate endothelial barrier function, and media flowed over the top of the membrane to promote nutrient exchange. The majority of scaffold-based chips use pre-formed 3D hydrogel cultures that subsequently introduce perfusion across the surface of the hydrogel structure (Bavli et al., 2016; Schepers et al., 2016; Aeby et al., 2018; Christoffersson et al., 2018; Yajima et al., 2018). Natarajan et al. (2022) modeled HCV infection in liver organoids encapsulated in basement membrane matrix and perfused on a commercially available chip, idenTx, from AIM Biotech (Natarajan et al., 2022). Alternatively, commercial supplier Hesperos utilized a nylon scaffold to separate cell types in their dual channel platform (Esch et al., 2015). Ma C. et al. (2016) devised a circular lobule-like structure using aggregated hepatic cords comprised of collagen-suspended hepatocytes and endothelial cells. Similar to the dielectrophoretic arrangement that Ho et al. (2013) used, Schütte et al. (2011) applied dielectrophoresis to assemble non-encapsulated 3D hepatic tissue constructs with PHHs and LSECs on ECM coated assembly gaps as the cell suspension flowed into the chip. These constructs were then perfused on both sides of the assembly gap after formation. 3D perfusion culture combines the advantages of a physiologically relevant 3D culture with the benefits of flow for nutrient exchange. The complexity of these designs, however, generally requires more lead time for device and culture assembly when compared to simpler 3D platforms without perfusion.

##### Perfused decellularized scaffolds

Sassi et al. (2021) recently published a platform to perfuse a recellularized rat liver dECM in a bioreactor. The authors cultured individual lobes of the reseeded liver under syringe pump perfusion and showed significant upregulation of hepatocyte maturation markers in the perfused culture over the course of 11 days (Sassi et al., 2021). The use of decellularized scaffolds for perfusion is a novel technique that may be an optimal method for sustaining a differentiated hepatocyte phenotype.

##### Bioprinted microfluidic platforms

Like bioprinted static culture, bioprinting offers additional spatial customizability for cell deposition. Bhise et al. (2016) instituted bioprinting as a means of encapsulated spheroid deposition, in a precise dot array pattern to create partitioned spheroid cultures. The authors bioprinted their cell-matrix suspension on to a glass slide, which was then sandwiched between two PDMS covered PMMA sheets to seal a perfusion channel (Bhise et al., 2016). Bioprinting is also useful for non-traditional matrices that are otherwise difficult to deposit. Lee et al. (2019) used dECM bioink to construct two separate layers, one with hepatocytes and one with endothelial cells. The layers were perfused on both sides to simulate blood flow across the endothelial layer and bile flow across the hepatocytes (Lee et al., 2019). Bioprinted platforms provide options for more sophisticated 3D tissue structures in microfluidic chips.

##### High-throughput and plate format perfusion platforms

While many of the platforms detailed above are not optimized for multiplexing and generating high-throughput data, several commercially available systems feature plate formats that are advantageous for adding biological replicates and testing a larger number of experimental conditions (Figure 2K). The PREDICT96 platform from Draper contains 24 individual microphysiological systems, pneumatically perfused, and arranged to fit a 96-well plate format. Each of the 24 chips has a top channel with collagen-suspended hepatocytes, a polycarbonate membrane, and a bottom channel for media flow (Tan et al., 2019). CN Bio’s LiverChip plate consists of 12 individual microphysiological systems: pneumatically driven media circulates up and through an ECM-coated polycarbonate scaffold with microchannels (Sarkar et al., 2015, 2017; Long et al., 2016; Rowe et al., 2018). Ortega-Prieto et al., 2018, 2019 infected PHHs with HBV for up to 22 days using the CN Bio chip, and demonstrated improved infection in the chip as compared to spheroid and traditional 2D cultures. MIMETAS retrofit their OrganoChip^®^ design to a 384 well plate with 40 individual culture chambers. Each chip has phaseguides that separate the matrix-encapsulated cells from rocking-induced gravity flow, but still permit nutrient exchange (Jang et al., 2015, 2018; Jang M. et al., 2019; Bircsak et al., 2021). Lena Biosciences’ design features a fiberglass scaffold with the option for matrix encapsulation. Cells are perfused bidirectionally via volume changes beneath a bottomless well insert that causes a rising and falling of media across the scaffold (Shoemaker et al., 2020). High-throughput plate designs translate complex physiologically relevant microsystems to a more familiar plate environment to improve their ease of use and efficacy for multi-replicate and multi-variable studies, relevant for studying diverse viral infection conditions simultaneously. A drawback when compared to other systems is that they require a more intricate assembly than some static culture systems, but this limitation holds true for nearly every perfusion device.

## Clinical implications for physiologically relevant platforms

**FIGURE 3:**
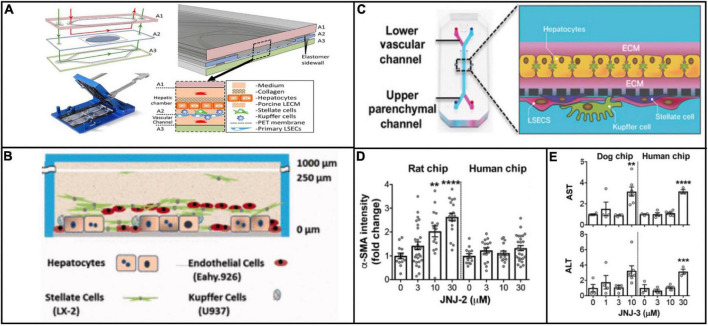
Examples of platforms that incorporate multiple cell types that are implicated in viral hepatitis pathogenesis. The platforms described in Li et al. (2018) (Reproduced with permissions from Li et al.) **(A)**, Vernetti et al. (2016) (Reproduced with permissions from Vernetti et al.) **(B)**, and Jang K. J. et al. (2019) (Reproduced with permissions from Jang et al.) **(C)** all include Kupffer cells and hepatic stellate cells in addition to hepatocytes and endothelial cells. The inclusion of all four cell types makes these platforms more effective for modeling a systemic response to viral infection. Jang et al. demonstrated that treatment of rat and human cells with a proprietary compound resulted in differential toxicity **(D)**, but treatment of dog and human cells with a different compound resulted in a similar toxicity profile **(E)**, evidence of the variation in responses between animal and human cells to different compounds. **P < 0.01, ***P < 0.001, and ****P < 0.0001.

Although the scope of applications for physiologically relevant systems has thus far been limited to *in vitro* modeling, it is important to consider what these platforms may provide as clinically predictive models. For viral hepatitis specifically, physiologically relevant microsystems hold great potential as tools to make clinical predictions about disease progression because these platforms incorporate numerous microenvironmental factors that are implicated in viral pathogenesis. For example, Kupffer cells and stellate cells recruit T cells and contribute to the inflammatory microenvironment during infection (Wang et al., 2013; Boltjes et al., 2014). Several different chip designs have incorporated these cells types (Vernetti et al., 2016; Li et al., 2018; Jang K. J. et al., 2019), and although they have not attempted viral hepatitis infection, these designs have obvious potential for enhancing the biological relevance of the cellular response to viral hepatitis infection (Figures 3A–C). Moreover, the notion of creating linked multi-organ platforms creates opportunity for an even larger systemic response to viral infection. Even though the three aforementioned platforms do not have T cells, they could be potentially linked with an immune chip where the recruitment of immune cells from a separate chamber could be studied.

Outside of the context of viral hepatitis, Emulate’s liver chip has already compared *in vitro* results against clinical data in terms of drug toxicity response. Jang et al. showed species-dependent differences in toxicity response to a Janssen compound that was discontinued during animal trials because of hepatoxicity in rats. These results were confirmed from with rat hepatocytes on the *in vitro* platform, but when the same experiment was conducted on human hepatocytes, no such toxic effect was observed (Figure 3D). This finding underscores obvious drawbacks of using animal models to test compounds intended for humans, in that some compounds are unnecessarily filtered out of contention due to species-dependent toxicity. Conversely, when testing a different proprietary compound that was discontinued due to hepatocellular necrosis in dogs, dog and human hepatocytes were aligned in their toxic response (Jang K. J. et al., 2019; Figure 3E). These findings highlight the potential for physiologically relevant microsystems to circumvent species-dependent discrepancies in response to drug treatment, making for more efficient compound screening and clinical translation.

## Conclusion

*In vitro* studies are needed to aid in the ascertainment of new knowledge on the mechanisms that govern the transition of viral hepatitis from acute to chronic infection and related pathogenic processes. A lack of readily available *in vivo* models necessitates the use of *in vitro* systems that can attain the level of physiological relevance that is observed *in vivo*. Importantly, the capacity to study hepatotrophic viruses on human cells using models that can be interrogated through sophisticated and informative molecular assays is required. The developments of physiologically relevant platforms are in their early stages in terms of their use as models of viral hepatitis infection; however, the available studies thus far have demonstrated significant improvements in infection efficacy and longevity as compared to conventional cell culture methods. These preliminary steps in establishing more robust infection models will undoubtedly create beneficial tools to study virus-host interactions and uncover novel insights about chronic viral infection and related mechanisms that would not otherwise be possible through the use of more rudimentary platforms. Some of the systems mentioned above have yet to be specifically utilized for viral hepatitis modeling, but their shared goal of improved hepatocyte function *in vitro* translates across all applications. Nonetheless, physiomimetic platforms still have a variety of barriers to overcome before becoming reliable models of viral infection. For example, making these models higher throughput with easy access to cultures in order to introduce and assay infection is critical for these platforms to compete with conventional 2D culture where these two elements are commonplace. Fortunately, the plethora of tools and models that now exist can serve as a solid foundation for improving platforms to study virus-host interaction. Many of these platforms are now available for use, but need to be introduced to viral applications.

## Author contributions

DM, DB, AA, and ET: manuscript concept, design and drafting of the manuscript. All authors contributed to the article and approved the submitted version.

## Abbreviations

HBV: hepatitis B virus
HCV: hepatitis C virus
HDV: hepatitis D virus
HEV: hepatitis E virus
HCC: hepatocellular carcinoma
2D: two-dimensional
3D: three-dimensional
cccDNA: covalently closed circular DNA
DAAs: direct acting antiviral agents
ECM: extracellular matrix
PHHs: primary human hepatocytes
iPSCs: induced pluripotent stem cells
NPCs: non-parenchymal cells
KCs: Kupffer cells
SCs: stellate cells
SARS-CoV2: severe acute respiratory syndrome coronavirus 2
NTCP: Na+-taurocholate cotransporting polypeptide
HLCs: hepatocyte-like cells
LBL: layer-by-layer
HUVECs: human umbilical vein endothelial cells
MPCC: micropatterned co-culture
dECM: decellularized ECM
PEGDA: polyethylene glycol diacrylate
RGD: arginine-glycine-aspartate
HF: hollow fiber
RWV: rotating wall vessel
MPCC: micropatterned co-cultures
PDMS: polydimethylsiloxane
PMMA: polymethyl methacrylate.
